# Metabolic Syndrome Status Changes and Cognitive Functioning: Insights from the Lifelines Cohort Study

**DOI:** 10.14283/jpad.2024.90

**Published:** 2024-05-28

**Authors:** I. Frentz, Sofia Marcolini, C. C. I. Schneider, M. A. Ikram, J. Mondragon, P. P. De Deyn

**Affiliations:** 1 Department of Neurology, University Medical Center Groningen, Groningen, the Netherlands Hanzeplein 1,; 2 Department of Epidemiology, Erasmus MC, Rotterdam, the Netherlands; 3 The Research School of Behavioural and Cognitive Neurosciences, University of Groningen, Ant. Deusinglaan 1, 9713 AV Groningen, The Netherlands; 4 Instituto de Neurobiología, Departamento de Neurobiología Conductual y Cognitiva, Laboratorio de Psicofisiología, Universidad Nacional Autónoma de México, Querétaro, 76230 Mexico; 5 Department of Psychology, Life-Span Human Senses Lab, San Diego State University, San Diego, California 92182 USA; 6 Laboratory of Neurochemistry and Behavior, University of Antwerp, 2610 Antwerp, Belgium

**Keywords:** Metabolic syndrome, Alzheimer’s disease, cognition, population-based study, risk factors

## Abstract

**Background:**

Metabolic syndrome is associated with increased risk of dementia. Yet, findings on how longitudinal development of metabolic syndrome status affects cognition remain controversial.

**Objectives:**

This study examines whether individuals with different changes in metabolic syndrome status differ in cognitive functioning. Additionally, the prevalence of metabolic syndrome within the Lifelines population-based study is investigated.

**Design:**

14609 Lifelines participants (mean age 60.8, 56.4% women) were divided into four groups based on their metabolic syndrome status changes between 2007–2013 (1) and between 2014–2017 (2): without metabolic syndrome (N=10863; absent at 1 and 2), de novo metabolic syndrome (N=1340; absent at 1 and present at 2), remitting metabolic syndrome (N=825; present at 1 and absent at 2), and persistent metabolic syndrome (N=1581; present at 1 and 2). ANCOVA models were employed to assess group differences in psychomotor function, visual attention, visual learning, and working memory assessed using the Cogstate Brief Battery.

**Results:**

Accounting for education, age, sex, and time between examinations, groups did not statistically differ in any of the four cognitive outcomes. The prevalence of metabolic syndrome within the Lifelines population increased with age and differed among men and women.

**Conclusion:**

Performance in psychomotor function, visual attention, visual learning, and working memory measured by the Cogstate Brief Battery did not differ between individuals with different changes in metabolic syndrome. The length of metabolic syndrome exposure was unknown, making our results exploratory and calling for future studies addressing this gap.

**Electronic Supplementary Material:**

Supplementary material is available for this article at 10.14283/jpad.2024.90 and is accessible for authorized users.

## Introduction

**M**etabolic syndrome is a cluster of pathologic conditions, characterized by hypertension, insulin resistance, abdominal obesity, and abnormal cholesterol or triglyceride levels (1, 2). The global prevalence of metabolic syndrome ranges from 12.5% to 31.4%, depending on diagnostic criteria, increases with age, and is highest in the Americas and Eastern Mediterranean regions (3, 4). In the Netherlands, prevalence is reported as 36% for men and 24% for women (5). There seems to be an association between metabolic syndrome and Alzheimer’s Disease, the most common cause of dementia, where the components of metabolic syndrome contribute to an increased risk of Alzheimer’s Disease (6–9). Shared pathogenic pathways, such as insulin resistance, neurohormonal activation, and inflammation, underscore the connection between metabolic syndrome and Alzheimer’s Disease (10). Furthermore, metabolic syndrome heightens the risk of cardiovascular disease, stroke, and diabetes (1).

Individuals exhibiting cardiometabolic traits and diseases related to insulin resistance were shown to perform worse in verbal and numerical reasoning and have slower processing speed (11). A study in a memory clinic population found that metabolic syndrome was associated with worse performance in executive function, attention, speed and visuoconstructive ability (12). Moreover, metabolic syndrome was also found to be associated with a large decline over a decade in percentual speed, a subcategory of processing speed, in midlife women (13). Although overall, the findings on the relationship between metabolic syndrome and cognition have been contradictory and inconsistent, with some studies finding no associations between metabolic syndrome and cognitive functioning (14, 15) and others finding some associations (2, 10, 16–18).

A potential explanation for these contradictory findings is that the conditions determining metabolic syndrome are highly variable and studies have mostly considered metabolic syndrome cross-sectionally, while studies considering changes in metabolic syndrome status in relation to cognition remain limited. In a prospective cohort study, participants with persistent metabolic syndrome had worse cognitive functioning than those without metabolic syndrome (19). Additionally, a population-based cohort study found higher dementia risk in participants with worsened metabolic syndrome, meaning absent at the first screening but present at the second, in a period of five years compared to those with persistent or improved metabolic syndrome, as well as higher risk in those with nonpersistent metabolic syndrome compared to those without metabolic syndrome (20). Notably, improvement in metabolic syndrome status was related to reduced occurrence of dementia (10).

The present study aims to investigate whether groups with different changes in metabolic syndrome status, namely, without, de novo, remitting, and persistent, differ in cognitive functioning. We expect the group with persistent metabolic syndrome to have the worst cognitive performance. Additionally, the prevalence of metabolic syndrome in the whole Lifelines sample is assessed.

## Methods

### Study design

Data was derived from the Lifelines Cohort Study, a large prospective population-based cohort study that started in 2006. The cohort is set in the northern part of the Netherlands and consists of a children’s cohort (aged 0–18 years), an adult cohort (18–65 years), and an elderly cohort (≥ 65 years at baseline). Lifelines collects physical, genetic, behavioral, psychological, and environmental data. Every 5 years participants have a follow-up visit with a physical examination, during which anthropometry and biological samples are collected. Between follow-up visits, participants are asked to fill out questionnaires (21). Currently, data collection for timepoint 3 is still ongoing. Therefore, data from timepoint 1a (2007–2013) and 2a (2014–2017) of the Lifelines database was available for analysis. The Lifelines timepoints 1a and 2a are referred throughout the manuscript as timepoint 1 and 2.

### Metabolic syndrome

Criteria for metabolic syndrome are defined by the National Cholesterol Education Program Adults Treatment Panel III classification (1). The Adults Treatment Panel III classification consists of the following 5 criteria: increased waist circumference (≥102 cm for men and ≥88 cm for women); plasma triglycerides ≥150 mg/dL (1.7 mmol/L); decreased plasma high-density lipoprotein (<40 mg/dL (1.03 mmol/L) in men and <50 mg/dL (1.29 mmol/L) in women); elevated blood pressure (systolic ≥130 mmHg or diastolic ≥85 mmHg) or medication for hypertension; fasting plasma glucose ≥100 mg/dL (5.6 mmol/L), or previously diagnosed type 2 diabetes. Metabolic syndrome is diagnosed when 3 or more of the aforementioned criteria are met. Participants were categorized into four groups based on metabolic syndrome status at timepoints 1 and 2. The first group were participants that were healthy at both timepoints: without metabolic syndrome; the second group was healthy at timepoint 1 but developed metabolic syndrome by timepoint 2: de novo metabolic syndrome; the third group had metabolic syndrome at timepoint 1 but was healthy at timepoint 2: remitting metabolic syndrome; and the fourth group had metabolic syndrome at both timepoints: persistent metabolic syndrome. Metabolic syndrome criteria at timepoints 1 and 2 within the four groups are shown in Supplementary Table 1, frequency of the components in the groups is shown in Supplementary Table 2. The mean time interval between timepoint 1 and 2 was 3.69 years (standard deviation = 0.93) and ranged between 0.91 and 8.25 years.

### Cognitive Performance

In the Lifelines cohort study, during assessment timepoint 2, cognition was measured using the Cogstate Brief Battery. This Battery was only administered at timepoint 2, so no data is available on participant’s baseline cognition at timepoint 1. The Cogstate Brief Battery is a computerized test that allows measurement of cognitive performance in large cohorts, it has good test-retest reliability and can be used in healthy and cognitively impaired patients (22, 23). Studies have shown that the Cogstate Brief Battery was capable of measuring cognitive decline in individuals who developed mild cognitive decline and individuals with Alzheimer’s Disease (24, 25). The Cogstate digital tests administered in Lifelines were four: the detection task, measuring psychomotor function and speed of processing; the identification task, measuring visual attention; the one-back task, measuring working memory and attention; and the one-card learning task, measuring visual learning and memory. A description of the tasks is given in Supplementary Table 3. The primary outcome for the detection and identification task is the speed of performance, expressed as the mean reaction time which is log10 transformed for normalization. For the one-back and one-card learning tasks the primary outcome is the accuracy of performance expressed as the proportion of correct answers which is arcsine square root transformed for normalization. These transformations result from the Cogstate outcome measures and have been determined by the developing team as indicated on their website. A lower score on detection and identification tasks and a higher score on one-back and one-card learning tasks indicate better cognitive performance. Only participants with complete and valid scores were retained according to the Cogstate research group completion criteria shown at: https://wiki.lifelines.nl/doku.php?id=cogstate.

### Covariables

For each participant, we obtained information on the highest level of completed education (classified into low, intermediate and high), and smoking habit (i.e., non-smoker or ever-smoker). Additionally, a pack-year variable was assessed; this was calculated by multiplying the number of packs of cigarettes smoked per day by the number of years a person has smoked. Physical activity levels were assessed through the Short Questionnaire to Evaluate Health-enhancing physical activity (SQUASH). This questionnaire, developed by the Dutch National Institute of Public Health and the Environment, serves to provide insights into habitual physical activity levels. The SQUASH gathers information on the frequency, type, duration, and intensity of activities during a typical week in the preceding months. The questions related to physical activity were distributed across four categories: commuting (which includes walking and bicycling to and from work), leisure time (involving walking, biking, gardening, odd jobs, and sports), household activities, and work/school-related activities. To quantify the intensity of the activities, the Ainsworth’s Compendium of Physical Activities was employed. This compendium assigns metabolic equivalent values (MET) to activities, categorizing them as either moderate (MET value of 4 to <6.5) or vigorous intensity (MET value of ≥6.5) (26). An average score of moderate and vigorous physical activity scores, calculated by multiplying the MET value with the duration (measured in minutes per week) of the activities (squash_sum_scores [Lifelines Wiki]) was used (26). The Lifelines Diet Score was used to determine relative diet quality. The Lifelines Diet Score is calculated based on the baseline 110-item food frequency questionnaire (the heart of the flower-leaf FFQ), per food group, the intake in grams per 1000 kcal is categorized into quintiles, awarded 0 to 4 points (negative groups scored inversely) and summed. This score is a food-based and evidence-based tool, its highest scores represent the most beneficial diet (27). All data analyzed was assessed at timepoint 1.

### Inclusion criteria

For the present study, we included 96880 participants. These participants had measurements at two timepoints (1 and 2) to establish metabolic syndrome diagnosis (waist circumference, fasting glucose or diabetes diagnosis, triglycerides, high-density lipoprotein, systolic and diastolic blood pressure measurements). We excluded 21856 participants that did not have cognition data available at timepoint 2. Next, we excluded 50711 participants that were younger than 50 years at timepoint 1. This age threshold was chosen considering that metabolic syndrome is more present with increasing age (6–9) and that its effect on cognitive functioning might only manifest at older age. 7097 participants did not have complete and valid results for the Cogstate Brief Battery at timepoint 2, leaving 17216 participants. Of these participants, we excluded 1298 participants that did not have information for all variables studied (education, gender, interval between timepoint 1 and 2) leaving 15918 participants. Finally, we excluded 1309 with extreme values for the cognitive outcomes (IQR method of outlier detection exceeding ± 1.5 interquartile range). The remaining sample consisted of 14609 participants (Figure 2).

**Figure 1:**
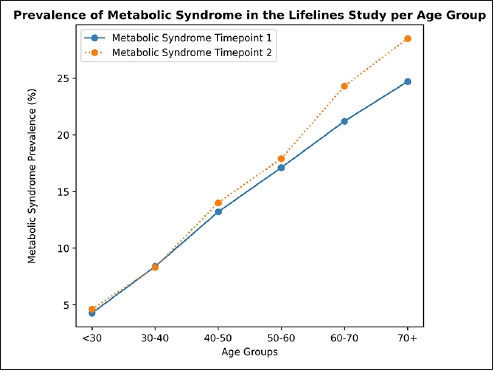
Prevalence of metabolic syndrome Prevalence of metabolic syndrome in the Lifelines population (N = 96880), shown for age groups. Metabolic syndrome was defined as 3 or more of the metabolic syndrome criteria being met. Sample per age group at timepoint 1: < 30 n = 11813, 30–40 n = 19290, 40–50 n = 34619, 50–60 n = 16386, 60–70 n = 11842, 70+ n = 2930. Sample per age group at timepoint 2: < 30 n = 6674, 30–40 n = 14763, 40–50 n = 29262, 50–60 n = 25821, 60–70 n = 14528, 70+ n = 5832. Prevalence of metabolic syndrome at timepoint 1 for the age groups ‘<30’, ‘30–40’, ‘40–50’, ‘50–60’, ‘60–70’, ‘70+’ respectively: 4.3%, 8.4%, 13.2%, 17.1%, 21.2%, 24.7%; prevalence at timepoint 2 for the age groups ‘<30’, ‘30–40’, ‘40–50’, ‘50–60’, ‘60–70’, ‘70+’ respectively: 4.6%, 8.3%, 14.0%, 17.9%, 24.3%, 28.5%.

**Figure 2:**
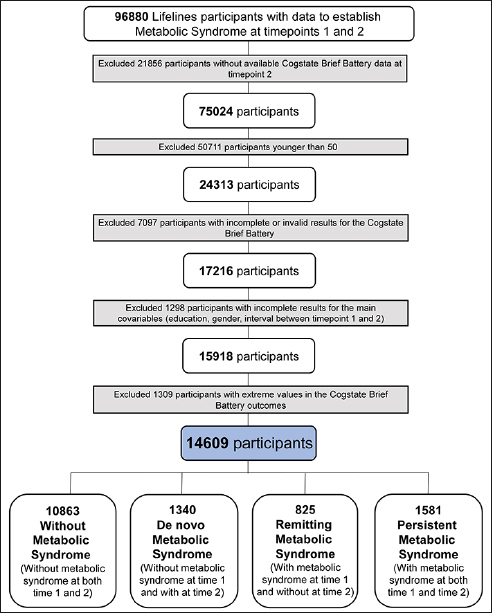
Inclusion flowchart Flowchart explaining the reasons for participants’ exclusion.

### Statistical analysis

The data was analyzed using R version 4.2.0 (Foundation for Statistical Computing, Vienna, Austria). Sample characteristics were described using percentages for categorical data, mean ± SD for normally distributed continuous data and median and interquartile range for skewed continuous data. Differences in the sample characteristics among groups were analyzed using ANOVAs for normally distributed continuous variables, chi-squares for categorical variables, and Kruskal-Wallis for non-normally distributed continuous variables. ANCOVA models were run to assess group differences in cognitive outcomes. For ease of comparison, for all cognitive measures, standardized scores were calculated by dividing the difference between the individual and mean test scores by the standard deviation. Assumptions were checked before running the ANCOVA models; for all outcomes skewness lied between +1 and −1. The ANCOVA models were run correcting for age at timepoint 2, sex, educational level, and time interval between measurement of metabolic syndrome at timepoint 1 and 2. A separate model was run for each cognitive test ending up with four models. All ANCOVA models were corrected for multiple comparisons using Bonferroni and considered a significance level of *α* = 0.05.

### Data availability

Anonymised data are available on reasonable request. Requests for access to the data reported in this paper can be directed to data managers of the Lifelines Cohort Study (research@lifelines.nl)

## Results

Characteristics of the study population are shown in Table 1. The mean age at baseline was 60.8 years (SD 6.1) and 8240 (56.4%) participants were women. At timepoint 1 20.0% (N = 2406) of participants had metabolic syndrome (persistent or remitting). Of these participants 5.7% (N = 825) improved from having metabolic syndrome to healthy at timepoint 2 (remitting), while 9.2% (N = 1340) of participants that were healthy at timepoint 1 had metabolic syndrome at timepoint 2 (de novo), making the total of participants with metabolic syndrome at timepoint 2 16.5% (N = 2921) (de novo or persistent).

**Table 1:** Population characteristics grouped by without metabolic syndrome, de novo metabolic syndrome, remitting metabolic syndrome and persistent metabolic syndrome

	**Without metabolic syndrome (n = 10863)**	**De novo metabolic syndrome (n = 1340)**	**Remitting metabolic syndrome (n = 825)**	**Persistent metabolic syndrome (n = 1581)**	**Overall (n = 14609)**	**p-value**
Age, years at timepoint 2	60.5 ± 6.1	61.4 ± 6.3	61.1 ± 6.1	61.8 ± 6.2	60.8 ± 6.1	<0.001^**^
Age range at timepoint 1	50–86	50–87	50–78	50–83	50–87	
Age range at timepoint 2	51–89	51–90	52–82	52–86	51–90	
Female sex	6278 (57.8%)	727 (54.3%)	419 (50.8%)	816 (51.6%)	8240 (56.4%)	<0.001^**^
Education level						<0.001^**^
Low	3439 (31.7%)	531 (39.6%)	318 (38.5%)	656 (41.5%)	4944 (33.8%)	
Intermediate	3474 (32.0%)	423 (31.6%)	278 (33.7%)	497 (31.4%)	4672 (32.0%)	
High	3950 (36.3%)	386 (28.8%)	229 (27.8%)	428 (27.1%)	4993 (34.2%)	
Cogstate Brief Battery score						
Detection task (median, IQR)	2.568 (2.487–2.666)	2.575 (2.498–2.673)	2.565 (2.495–2.670)	2.584 (2.506–2.686)	2.570 (2.490–2.669)	
Identification task (median, IQR)	2.695 (2.654–2.744)	2.699 (2.657–2.746)	2.700 (2.658–2.747)	2.706 (2.663–2.752)	2.697 (2.655–2.745)	
One-back task (median, IQR)	2.919 (2.862–2.981)	2.918 (2.861–2.976)	2.925 (2.861–2.986)	2.925 (2.869–2.982)	2.923 (2.863–2.981)	
One-card learning task (median, IQR)	0.964 (0.896–1.033)	0.964 (0.886–1.033)	0.951 (0.886–1.033)	0.951 (0.886–1.019)	0.964 (0.891–1.033)	
Pack-years (median, IQR) at timepoint 1	8.8 (3.5–18.0)	12.0 (5.3–22.6)	13.5 (5.5–23.7)	15.5 (7.0–26.4)	10.0 (4.0–19.8)	<0.001^**^
Smoking habit (non-smokers) at timepoint 1	3524 (35.9%)	324 (27.3%)	201 (27.4%)	382 (27.1%)	4431 (33.7%)	<0.001^**^
Diet score at timepoint 1	26.68 ± 5.74	25.73 ± 5.80	25.45 ± 5.85	24.92 ± 5.78	26.34 ± 5.79	<0.001^**^
Physical activity score (median, IQR) at timepoint 1	726.2 (300.0–1440.0)	595.0 (225.0–1327.5)	565.0 (187.5–1237.5)	510.0 (150.0–1110.0)	675.0 (300.0–1383.8)	<0.001^**^
Interval assessment timepoint 1 and 2	3.67 ± 0.93	3.75 ± 0.91	3.71 ± 0.96	3.66 ± 0.95	3.69 ± 0.93	0.128

SD = standard deviation; IQR = interquartile range; de novo metabolic syndrome = participants without metabolic syndrome at timepoint 1 and with metabolic syndrome at timepoint 2; remitting metabolic syndrome = participants with metabolic syndrome at timepoint 1 and not at timepoint 2; persistent metabolic syndrome = participants with metabolic syndrome at both 1 and 2. Data are presented as frequency (%) for categorical variables and mean ± SD for continuous variables unless indicated otherwise. Complete data for pack-years (5913 missing), diet score (990 missing), and physical activity (1016 missing) was not available for all participants.

The prevalence of metabolic syndrome at timepoint 1 and 2 in the Lifelines population, consisting of 96880 individuals, is shown in Figure 1. Metabolic syndrome prevalence increased with age from 4.3% under the age of 30 to 24.7% after age 70 at timepoint 1, and from 4.6% under the age of 30 to 28.5% after age 70 at timepoint 2 (Figure 1). We also show in Supplementary Figure 1 a higher prevalence of metabolic syndrome in men compared to women. The most common criteria of metabolic syndrome in all groups are waist circumference, fasting glucose and blood pressure (frequencies of each component in each group are reported in Supplementary Table 2). Correlation matrices between all metabolic syndrome components and the cognitive outcomes are provided in the Supplementary Material (Supplementary Figure 2).

Group differences in sample characteristics are shown in Table 1. The group without metabolic syndrome was the youngest, had the highest percentage of females, the most higher educated and non-smokers participants, and the highest physical activity score. It also had better diet than all the other three groups and the lowest number of pack-year.

### Metabolic syndrome and cognition

Table 2 shows the results for group differences in the detection, identification, one-back, and one-card learning tasks in the entire study population. The pairwise comparisons of the cognitive outcomes between metabolic syndrome groups are shown in Table 3. We find no significant group differences for any of the four cognitive outcomes when running the ANCOVA models (p > 0.05). In a sensitivity analysis run without outlier removal, similar results are found.

**Table 2:** Group differences in cognitive performance

**Dependent variables**	**Without metabolic syndrome (n = 10863)**	**De novo metabolic syndrome (n = 1340)**	**Remitting metabolic syndrome (n = 825)**	**Persistent metabolic syndrome (n = 1581)**
Detection task	0.107 (0.094; 0.120)	0.102 (0.064; 0.139)	0.082 (0.035; 0.130)	0.144 (0.109; 0.178)
Identification task	0.167 (0.153; 0.180)	0.154 (0.115; 0.192)	0.182 (0.133; 0.235)	0.216 (0.180; 0.252)
One-card learning task	0.158 (0.143; 0.173)	0.103 (0.060; 0.146)	0.159 (0.104; 0.214)	0.151 (0.111; 0.191)
One-back task	0.089 (0.073; 0.104)	0.084 (0.040; 0.127)	0.067 (0.012; 0.122)	0.049 (0.009; 0.089)

Values depict adjusted mean differences with 95% confidence intervals. Models were adjusted for age at timepoint 2, sex, educational level, and time interval between measurement of metabolic syndrome at timepoint 1 and 2

**Table 3:** Pairwise comparisons of cognitive performance between metabolic syndrome groups

**Dependent variables**	**Detection task**	**Identification task**	**One-card learning task**	**One-back task**
Pairwise comparison Without and De novo	0.005 (−0.048; 0.059)	0.013 (−0.042; 0.068)	0.055 (−0.007; 0.117)	0.005 (−0.058; 0.067)
Pairwise comparison Without and Remitting	0.025 (−0.042; 0.092)	−0.015 (−0.084; 0.054)	−0.001 (−0.078; 0.076)	0.022 (−0.056; 0.099)
Pairwise comparison Without and Persistent	−0.036 (−0.086; 0.014)	−0.049 (−0.101; 0.002)	0.007 (−0.050; 0.065)	0.039 (−0.019; 0.097)
Pairwise comparison De novo and Remitting	0.019 (−0.062; 0.101)	−0.028 (−0.112; 0.056)	−0.056 (−0.150; 0.038)	0.017 (−0.078; 0.112)
Pairwise comparison De novo and Persistent	−0.042 (−0.110; 0.027)	−0.062 (−0.133; 0.008)	−0.048 (−0.127; 0.031)	0.035 (−0.045; 0.114)
Pairwise comparison Remitting and Persistent	−0.061 (−0.141; 0.018)	−0.034 (−0.116; 0.047)	0.008 (−0.083; 0.100)	0.018 (−0.074; 0.109)

Values depict adjusted mean differences with 95% confidence intervals.

## Discussion

In this large population-based study we find that older adults with different changes in metabolic syndrome status do not differ in cognitive performance in four subtasks of the Cogstate Brief Battery measuring psychomotor function, visual attention, visual learning, and working memory. The four groups examined were participants without metabolic syndrome, remitting metabolic syndrome, de novo metabolic syndrome and persistent metabolic syndrome. We find that at baseline the group with persistent metabolic syndrome has lower physical activity, worse diet habits, and a higher percentage of smokers. In the whole Lifelines sample of 96880 participants studied here, the prevalence of metabolic syndrome increases with age from 4.3% under the age of 30 to 24.7% after age 70 at timepoint 1, and from 4.6% under the age of 30 to 28.5% after age 70 at timepoint 2 and is higher in males for all age groups, except from the age of 60 onwards.

The null results found in the current study show that the group with persistent metabolic syndrome has worse cognitive performance compared to those with remitting, de novo, and without metabolic syndrome, although these differences were not significant. Our results do not support previous studies that found worse cognitive performance in individuals with persistent metabolic syndrome compared to those without or with intermittent metabolic syndrome (19, 20). A pooled meta-analysis showed an increased risk of Alzheimer’s Disease in individuals with metabolic syndrome, however, this increased risk of Alzheimer’s Disease was largely driven by two large retrospective studies (28). A study of a large Singaporean cohort also showed that participants with metabolic syndrome had an increased risk of developing dementia (29). Another previous study has also found that women with persistent and intermittent metabolic syndrome had lower physical health-related quality of life compared to those without (30). In contrast, a meta-analysis of clinical and population-based studies found that the pooled effect of metabolic syndrome on dementia and Alzheimer’s Disease incidence was minimal and not statistically significant (17). Metabolic syndrome instead increased the incidence of pure vascular dementia and in patients with mild cognitive impairment it seemed to increase the probability of progression to dementia (17). In a Korean cohort, an increase in waist circumference in older people was instead associated with a lower risk for Alzheimer’s Disease (10).

The period between the two metabolic syndrome assessment periods might have been too short to detect the impact of metabolic syndrome on cognitive function. Furthermore, the choice of participants who were fifty years old at the start of the study may have been too early to identify these distinctions. As shown by our prevalence results, the findings also indicate that the Lifelines cohort is a relatively healthy cohort compared to the prevalence shown in the Netherlands in a previous study, at least in men (5.)

Considering previous studies using the Cogstate Brief Battery (31, 32), although in different populations, the score results are comparable. The lack of effects found also highlight the complexity and multifactorial nature of the phenomena under investigation, suggesting that other variables not considered in the study might affect the characterization of cognition in these groups or that after all, our characterization of metabolic syndrome groups and the outcomes used, do not reflect clinically meaningful differences in cognition.

Our results, not showing an effect of metabolic syndrome on cognition, do not exclude that this condition can still have effects on brain function; in our study it might also have been too early to already detect differences in cognition. Several mechanisms have been proposed to describe the effect of metabolic syndrome on brain function. One of these suggests that, in individuals with metabolic syndrome, cerebral vascular reactivity, fundamental to maintaining energy-dependent processes, clearing metabolic waste, and involving capillary recruitment, is dysfunctional (33). This would be the result of insulin resistance and obesity-related inflammation which impacts the microvasculature (33). In metabolic syndrome, insulin resistance is also a consequence of sustained inflammation in peripheral tissue; insulin has a neuroprotective effect and by regulating synaptic plasticity it is fundamental for optimal cognitive functioning (6, 34). Previous neuroimaging studies have found that the cognitive underperformance seen in metabolic syndrome was mediated by white matter abnormalities measured with diffusion tensor imaging (35) and by cerebral blood flow in a study measuring immediate memory performance (36). In this latter study, results suggest that the reduced cerebral blood flow seen in metabolic syndrome was related more to arterial disease than to a decrease in metabolic function; although their study did not show structural brain alterations in individuals with metabolic syndrome at midlife (36). Altered resting state functional connectivity (37) and increased white matter hyperintensities volume (14) have also been observed in individuals with metabolic syndrome. Several studies support the idea that brain damage seen in metabolic syndrome is likely vascular in nature (33), and might be related to blood-brain barrier functions although the mechanisms underlying this potential relationship still remain unclear (38). The effects of metabolic syndrome on the vasculature and circulation, including reduced capillary density, increased arterial stiffness, and decreased cerebral blood flow, have been summarized elsewhere (39). Previous research in adolescents with metabolic syndrome and obesity also showed white matter abnormalities, and also a reduction in retinal arteriolar width, a biomarker for cerebral microvascular integrity (40).

While the main aim of the current study was not that of examining the relationship between modifiable risk factors and cognition in this population, we do see that groups with metabolic syndrome status change show differences in lifestyle factors. Although beyond the scope of this paper, a recent meta-analysis studying the association between the American Heart Association Cardiovascular Health metrics (smoking, diet, physical activity, body mass index, blood pressure, total cholesterol, and fasting glucose) and incident dementia found that individuals with a more favorable cardiovascular health profile had a stronger reduction in dementia risk than those with a poor or intermediate cardiovascular health profile. This meta-analysis suggests that improving cardiovascular health can substantially reduce the risk of dementia (41). Potential treatments could focus on, among others, two important common pathological pathways of metabolic syndrome and Alzheimer’s Disease: inflammation and insulin resistance. Neuroinflammation is an important driver in the pathophysiology of Alzheimer’s Disease, as the innate immune cells of the brain react to systemic inflammatory events rapidly, a response which is increased in elderly and diseased brains (42). Metabolic syndrome and the individual components that make up metabolic syndrome are associated with chronic systemic inflammation. Some studies have shown that dietary interventions were able to reduce inflammation (43–45), supporting lifestyle intervention as potential treatment. Modulation of insulin resistance in the brain has potential as a therapy to improve memory and Alzheimer’s Disease pathology, several therapeutic approaches that modulate insulin resistance in the brain are being investigated (46). Short-term administration of intranasal insulin improved episodic memory in patients with Mild Cognitive Impairment and Alzheimer’s Disease, and a placebo-controlled phase 2 and 3 trial showed a better cognitive performance in the insulin treatment group (46). Another promising therapeutic option is the GLP-1 receptor agonist, which increases peripheral insulin sensitivity and leads to visceral fat loss.

There are several strengths to this study. First, it was conducted in a large population-based cohort with standardized protocols and quality control for data collection. Next, metabolic syndrome was considered present when three or more of the criteria were met, this was used instead of separate predictors such as diabetes or obesity, as the individual components of metabolic syndrome are intricately interrelated and therefore cannot be studied in isolation in an epidemiological study. Additionally, the use of the Cogstate Brief Battery allowed for the assessment of cognitive performance in four different cognitive domains. Our study also had some limitations. The true length of metabolic syndrome exposure was unknown and the relatively short time between the two assessments might have influenced the impact of metabolic status on cognition, making our results rather exploratory. Additionally, the vast majority of participants (>98%) were of European ancestry, rendering results potentially less generalizable to other ancestries. For future research, it may be beneficial to investigate whether the severity of metabolic syndrome affects cognitive performance. The severity of metabolic syndrome could be assessed by using a continuous metabolic severity score, such as that developed by Gurka et al., (47). This continuous metabolic score allows researchers to examine the effect of temporal changes in metabolic syndrome. It would also allow researchers to better examine whether at any point in time metabolic syndrome is associated with cognitive functioning. Future research should also investigate whether the inflammatory markers associated with metabolic syndrome interact with cognitive performance and decline. Other potential confounding factors should be considered such as thyroid function and fatigue, which all affect cognitive performance. Baseline cognition, at timepoint 1, was unknown. The aim of the current study was to investigate whether cognition at timepoint 2 differed in groups with changes in metabolic syndrome status, while change in cognition was not evaluated. Future studies should also consider including baseline cognition in the examinations and evaluate how metabolic status changes affect longitudinal changes in cognition.

In conclusion, groups without metabolic syndrome, with de novo metabolic syndrome, with remitting metabolic syndrome, and with persistent metabolic syndrome did not differ in cognitive performance measured by the Cogstate in a large population-based Dutch cohort. Longitudinal studies with multiple cognitive assessments’ follow-ups are needed to confirm the null effect of temporal changes of metabolic syndrome on cognitive performance, also using other cognitive outcomes. Strategies aimed at the treatment and prevention of metabolic syndrome might still benefit cognition and slow down cognitive decline. Future studies should also take into consideration the age at which assessments took place and participant’s sex considering the trends found in metabolic syndrome prevalence in our cohort.

## Supplementary Material

Supplementary material, approximately 661 KB.
