# Supplementary material for: Metabolic Syndrome Status Changes and Cognitive Functioning: Insights from the Lifelines Cohort Study
Source: J Prev Alzheimers Dis. 2024 May 28;11(5):1283–90. doi: 10.14283/jpad.2024.90 (PMC11436447; doi:10.14283/jpad.2024.90)

**Supplementary Material**

**Supplementary Table 1**  Variables used for the metabolic syndrome criteria at timepoint 1 and 2 grouped by without metabolic syndrome, de novo metabolic syndrome, remitting metabolic syndrome and persistent metabolic syndrome.

| ***Metabolic syndrome criteria at timepoint 1*** | | | | | | |
| --- | --- | --- | --- | --- | --- | --- |
|  | **Without metabolic syndrome**  **(n = 10863)** | **De novo metabolic syndrome**  **(n = 1340)** | **Remitting metabolic syndrome**  **(n = 825)** | **Persistent metabolic syndrome**  **(n = 1581)** | **Overall**  **(n = 14609)** |  |
| **Waist circumference (cm, sitting)** | 89.3 (± 10.1) | 97.8 (± 9.8) | 101.3 (± 9.6) | 104.8 (± 10.3) | 92.4 (± 11.5) | **0.000**** |
| **Triglycerides (mmol/L; median, IQR)** | 0.9 (0.7-1.2) | 1.3 (1.1-1.6) | 1.8 (1.2-2.2) | 1.9 (1.4-2.6) | 1.1 (0.8-1.5) | **0.000**** |
| **Plasma high-density lipoprotein (mmol/L)** | 1.7 (± 0.4) | 1.4 (± 0.3) | 1.3 (± 0.3) | 1.2 (± 0.3) | 1.6 (± 0.4) | **0.000**** |
| **Fasting glucose (mmol/L; median, IQR)** | 4.9 (4.6-5.2) | 5.3 (5.0-5.5) | 5.6 (5.0-5.9) | 5.8 (5.3-6.3) | 5.0 (4.7-5.4) | **0.000**** |
| **Systolic blood pressure (mmHg)** | 126.6 (± 15.3) | 131.2 (± 15.3) | 137.7 (± 13.7) | 138.8 (± 14.0) | 128.9 (± 15.7) | **0.000**** |
| **Diastolic blood pressure (mmHg)** | 74.8 (± 9.3) | 76.3 (± 9.2) | 79.8 (± 9.2) | 79.5 (± 9.5) | 75.7 (± 9.5) | **<0.001**** |
| ***Metabolic syndrome criteria at timepoint 2*** | | | | | | |
|  | **Without metabolic syndrome**  **(n = 10863)** | **De novo metabolic syndrome**  **(n = 1340)** | **Remitting metabolic syndrome**  **(n = 825)** | **Persistent metabolic syndrome**  **(n = 1581)** | **Overall**  **(n = 14609)** |  |
| **Waist circumference (cm, sitting)** | 89.3 (± 10.3) | 100.8 (± 10.0) | 98.0 (± 10.2) | 105.0 (± 10.7) | 92.5 (± 11.8) | **0.000**** |
| **Triglycerides (mmol/L; median, IQR)** | 1.0 (0.8-1.3) | 1.8 (1.3-2.2) | 1.3 (1.0-1.6) | 1.9 (1.4-2.5) | 1.1 (0.8-1.5) | **0.000**** |
| **Plasma high-density lipoprotein (mmol/L)** | 1.7 (± 0.4) | 1.3 (± 0.4) | 1.4 (± 0.3) | 1.2 (± 0.3) | 1.6 (± 0.4) | **0.000**** |
| **Fasting glucose (mmol/L; median, IQR)** | 4.9 (4.7-5.2) | 5.6 (5.2-5.9) | 5.3 (4.9-5.5) | 5.9 (5.4-6.7) | 5.1 (4.7-5.5) | **0.000**** |
| **Systolic blood pressure (mmHg)** | 130.7 (± 16.0) | 141.4 (± 14.1) | 136.1 (± 16.4) | 144.1 (± 15.1) | 133.5 (± 16.5) | **0.000**** |
| **Diastolic blood pressure (mmHg)** | 74.6 (± 9.3) | 77.9 (± 9.0) | 76.1 (± 9.6) | 78.7 (± 9.4) | 75.4 (± 9.4) | **<0.001**** |

SD = standard deviation; IQR = interquartile range; de novo metabolic syndrome = participants without metabolic syndrome at 1 and with metabolic syndrome at 2; remitting metabolic syndrome = participants with metabolic syndrome at 1 and not at 2; persistent metabolic syndrome = participants with metabolic syndrome at both 1 and 2. Data are presented as mean ± SD unless indicated otherwise.

**Supplementary Table 2**  Percentage of metabolic syndrome criteria at timepoint 1 and 2 grouped by without metabolic syndrome, de novo metabolic syndrome, remitting metabolic syndrome and persistent metabolic syndrome.

| ***Metabolic syndrome criteria at timepoint 1*** | | | | |
| --- | --- | --- | --- | --- |
|  | **Without metabolic syndrome**  **(n = 10863)** | **De novo metabolic syndrome**  **(n = 1340)** | **Remitting metabolic syndrome**  **(n = 825)** | **Persistent metabolic syndrome**  **(n = 1581)** |
| **Waist circumference** | 3163 (29.1%) | 806 (60.2%) | 700 (84.9%) | 1415 (89.5%) |
| **Triglycerides** | 746 (6.9%) | 269 (20.1%) | 494 (59.9%) | 986 (62.4%) |
| **Plasma high-density lipoprotein** | 291 (2.7%) | 141 (10.5%) | 286 (34.7%) | 746 (47.2%) |
| **Fasting glucose** | 981 (9.0%) | 331 (24.7%) | 453 (54.9%) | 1079 (68.3%) |
| **Blood pressure** | 4253 (39.2%) | 613 (45.8%) | 705 (85.5%) | 1285 (81.3%) |
| ***Metabolic syndrome criteria at timepoint 2*** | | | | |
|  | **Without metabolic syndrome**  **(n = 10863)** | **De novo metabolic syndrome**  **(n = 1340)** | **Remitting metabolic syndrome**  **(n = 825)** | **Persistent metabolic syndrome**  **(n = 1581)** |
| **Waist circumference** | 3232 (29.8%) | 1107 (82.6%) | 458 (55.5%) | 1418 (89.7%) |
| **Triglycerides** | 762 (7.0%) | 804 (60.0%) | 147 (17.8%) | 1002 (63.4%) |
| **Plasma high-density lipoprotein** | 303 (2.8%) | 465 (34.7%) | 71 (8.6%) | 757 (47.9%) |
| **Fasting glucose** | 1039 (9.6%) | 804 (60.0%) | 213 (25.8%) | 1121 (70.9%) |
| **Blood pressure** | 5324 (49.0%) | 1172 (87.5%) | 497 (60.2%) | 1382 (87.4%) |

SD = standard deviation; IQR = interquartile range; de novo metabolic syndrome = participants without metabolic syndrome at 1 and with metabolic syndrome at 2; remitting metabolic syndrome = participants with metabolic syndrome at 1 and not at 2; persistent metabolic syndrome = participants with metabolic syndrome at both 1 and 2. Data are presented as percentage of the variable being present (or positive, namely meeting the diagnostic criteria described in the manuscript) in each group.

**Supplementary Table 3** Cogstate Brief Battery tasks description.

| **Test** | **Domain** | **Task** | **Primary outcome measure** |
| --- | --- | --- | --- |
| **Detection task** | Psychomotor function and speed of processing | Participants focus on the card in the center of the screen and follow the rule: “Has the card turned over?” Participants were instructed to press the “Yes” key as soon as the card turned. | Reaction time in milliseconds; normalized using log10 transformation.  The task ended after 35 correct trials. |
| **Identification task** | Visual attention | Participants focus on the card in the center of the screen and answer the question: “Is this card red?” Participants were instructed to press the “Yes” key if the card was and the “No” key if it was not. | Reaction time in milliseconds; normalized using log10 transformation.  The task ended after 30 correct responses. |
| **One-back task** | Working memory and attention | Participants focus on the card in the center of the screen and answer the question: “Is this the same card as the one of the previous trial?” Participants were instructed to press the “Yes” key if the answer was yes and the “No” key if the answer was no. | Proportion of correct answers; normalized using arcsine transformation.  The task ended after 30 correct trials. |
| **One-card learning task** | Visual learning and memory | Participants focus on the card in the center of the screen and answer the question: “Have you seen this card before in this task?” Participants were instructed to press the “Yes” key if the answer was yes and the “No” key if the answer was no. | Proportion of correct answers; normalized using arcsine transformation  The task ended after 42 trials. |

**Supplementary Figure 1. Prevalence of Metabolic Syndrome in Males and Females**


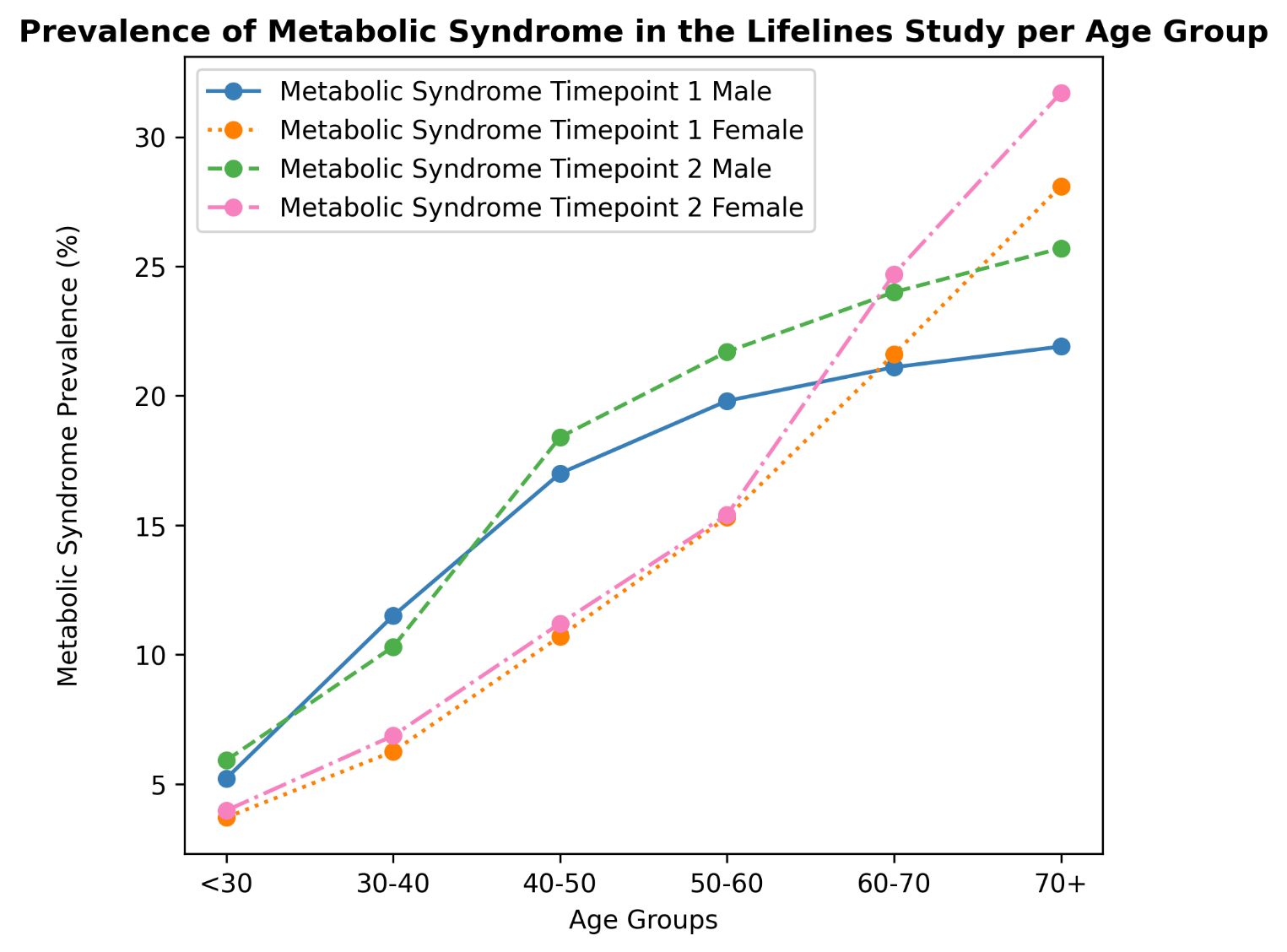


***Note.*** Sample per age group at timepoint 1 males: < 30 n = 4666, 30-40 n = 7903, 40-50 n = 13855, 50-60 n = 6774, 60-70 n = 5481, 70+ n = 1411. Sample per age group at timepoint 2 males: < 30 n = 2350, 30-40 n = 6387, 40-50 n = 11645, 50-60 n = 10526, 60-70 n = 6366, 70+ n = 2816. Sample per age group at timepoint 1 females: < 30 n = 7032, 30-40 n = 11247, 40-50 n = 20501, 50-60 n = 9516, 60-70 n = 6277, 70+ n = 1482. Sample per age group at timepoint 2 females: < 30 n = 4256, 30-40 n = 8246, 40-50 n = 17399, 50-60 n = 15125, 60-70 n = 8077, 70+ n = 2952. 735 cases have missing values for sex and have therefore not been included in this analysis.

**Complete data on metabolic syndrome prevalence.**

1. **For males:**

At timepoint 1 for the age groups '<30', '30-40', '40-50', '50-60', '60-70', '70+' respectively: 5.2%, 11.5%, 17.0%, 19.8%, 21.1%, 21.9%

At timepoint 2 for the age groups '<30', '30-40', '40-50', '50-60', '60-70', '70+' respectively: 5.9%, 10.3%, 18.4%, 21.7%, 24.0%, 25.7%

1. **For females:**

At timepoint 1 for the age groups '<30', '30-40', '40-50', '50-60', '60-70', '70+' respectively: 3.7%, 6.3%, 10.7%, 15.3%, 21.6%, 28.1%

At timepoint 2 for the age groups '<30', '30-40', '40-50', '50-60', '60-70', '70+' respectively: 4.0%, 6.9%, 11.2%, 15.4%, 24.7%, 31.7%

**Correlation matrices of the separate metabolic syndrome components at timepoint 1a (also referred to as timepoint 1 in the manuscript) and timepoint 2a (also referred to as timepoint 2 in the manuscript) and cognitive outcomes.**

**In the whole sample:**


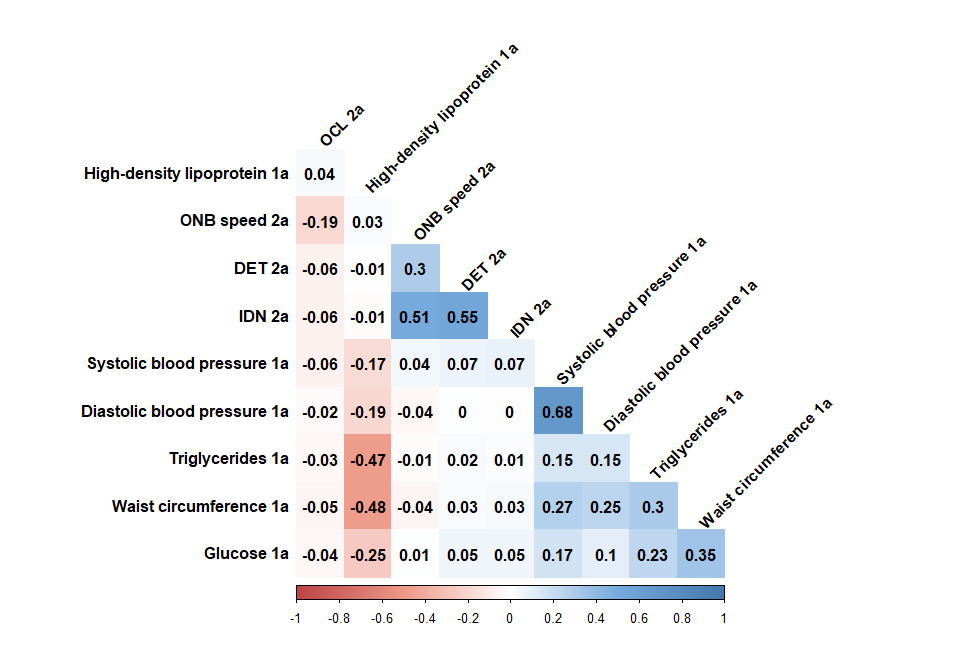


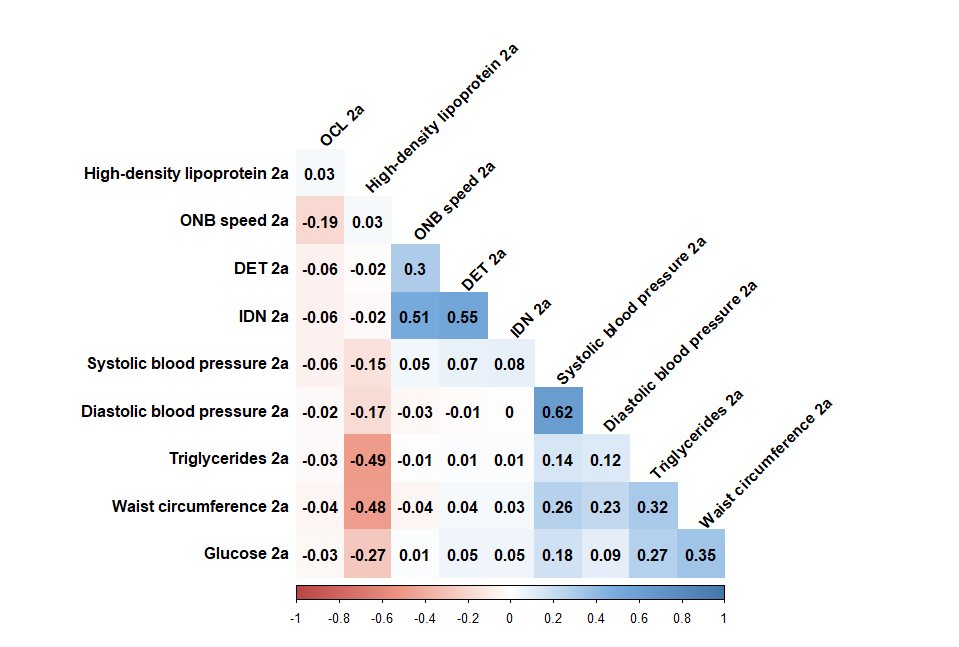


**In the without metabolic syndrome group:**


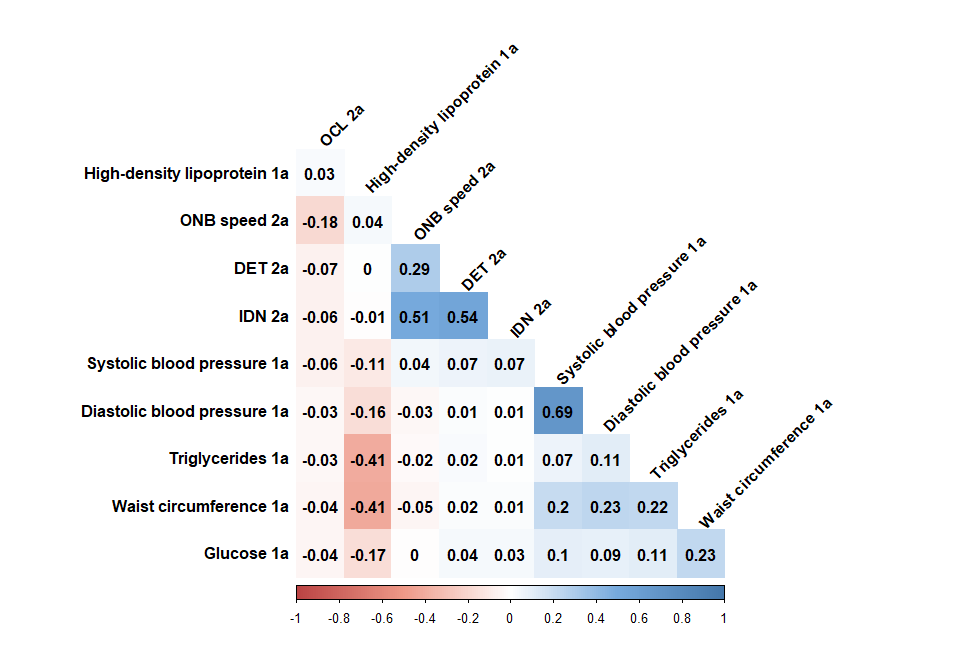


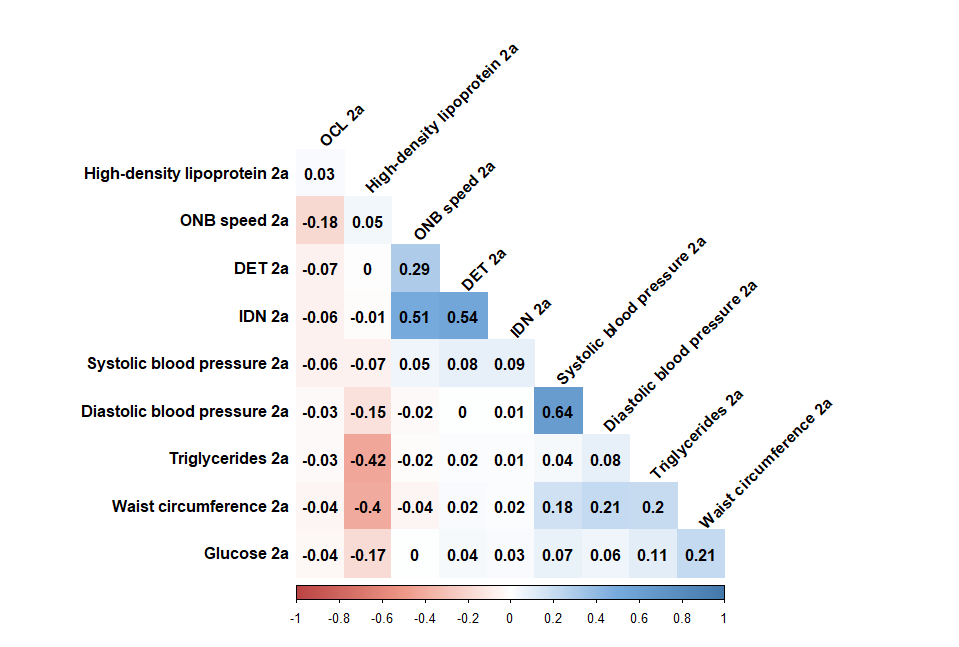


**In the de novo metabolic syndrome group:**


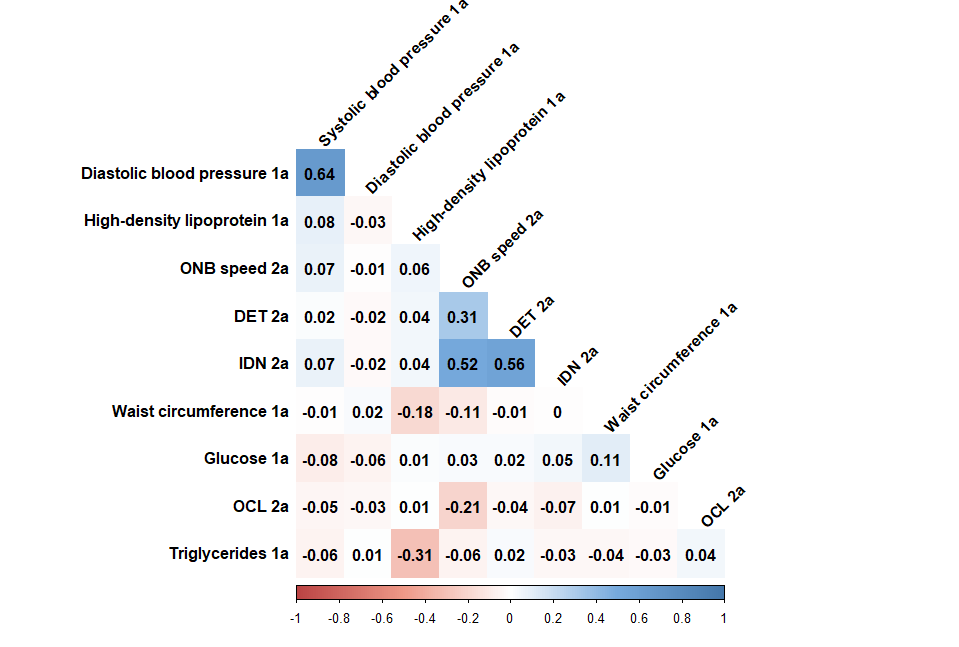


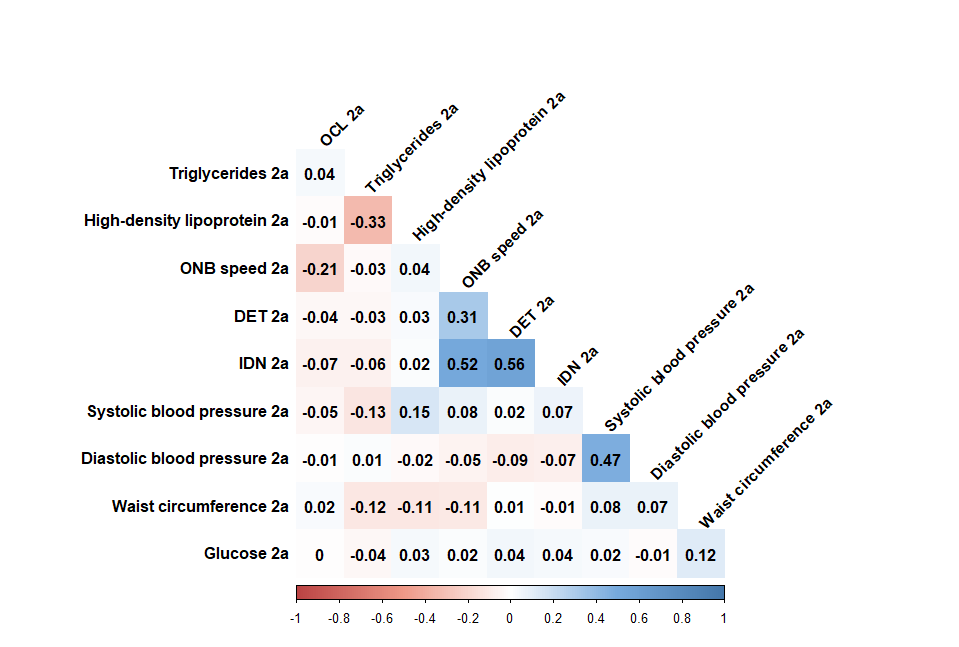


**In the remitting metabolic syndrome group:**


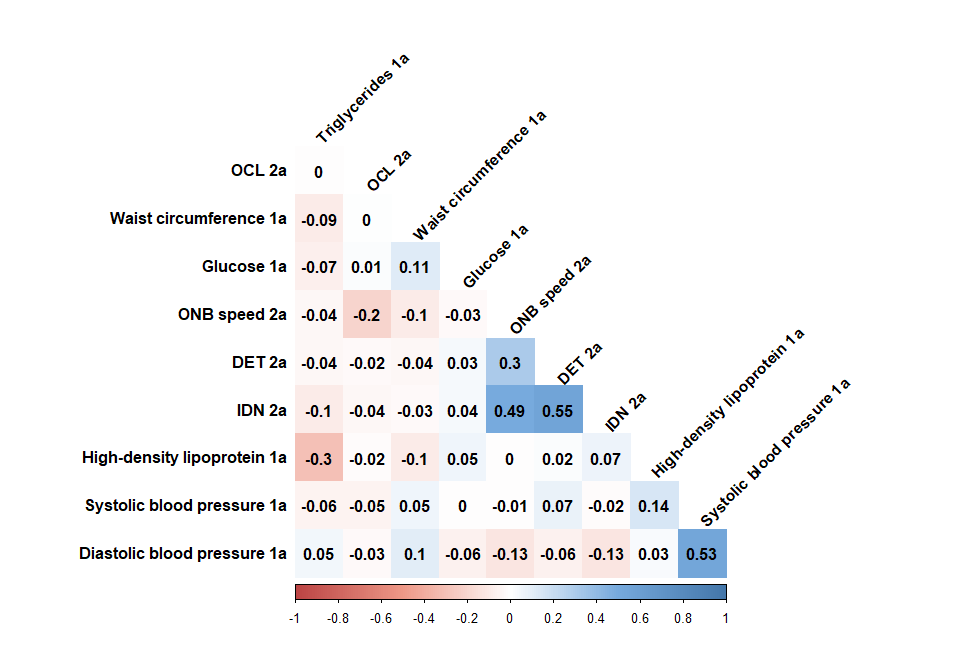


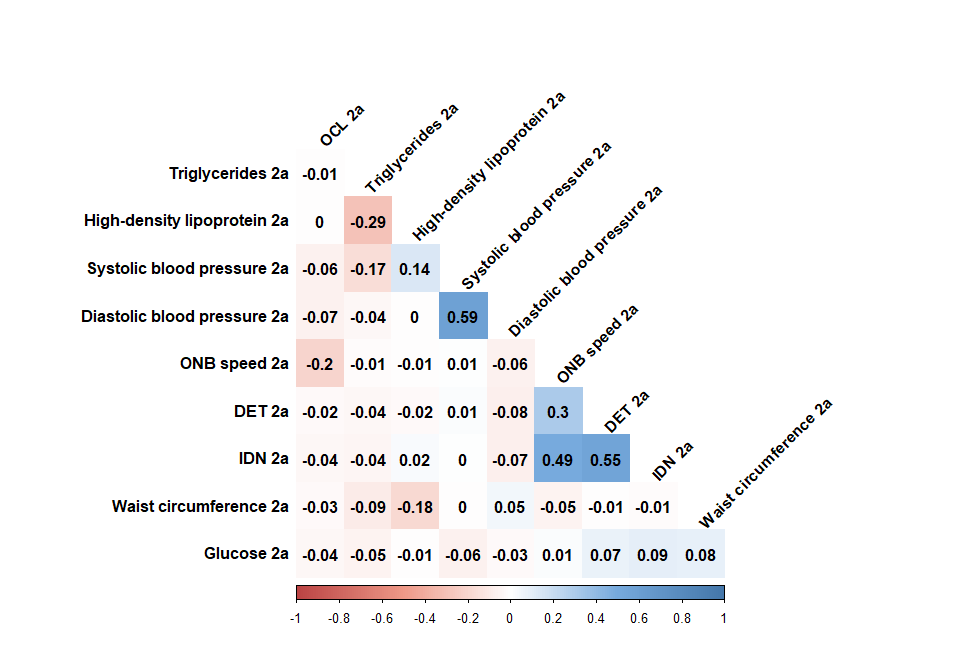


**In the persistent metabolic syndrome group:**


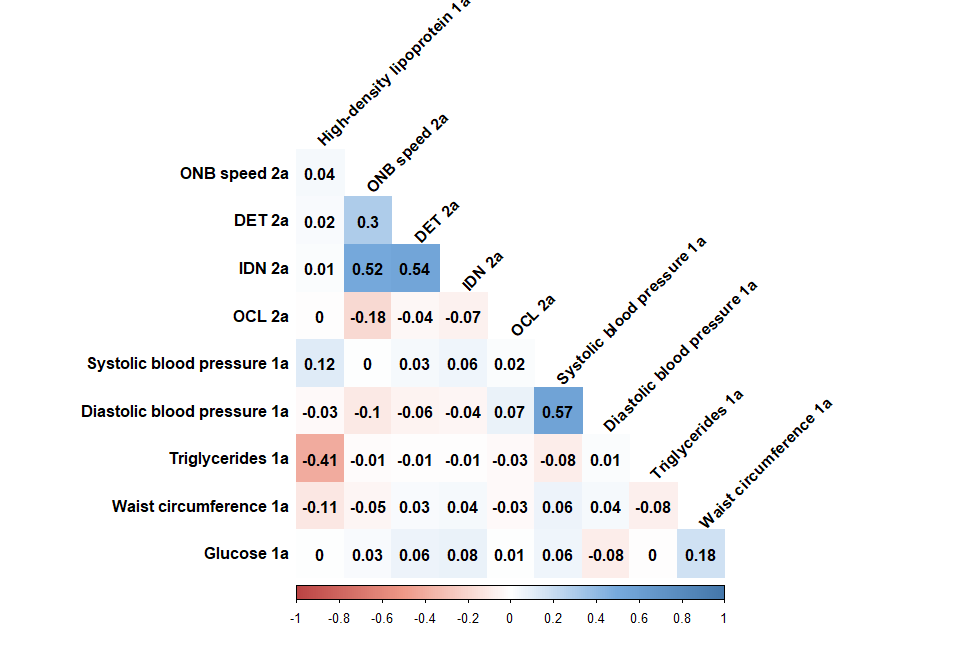


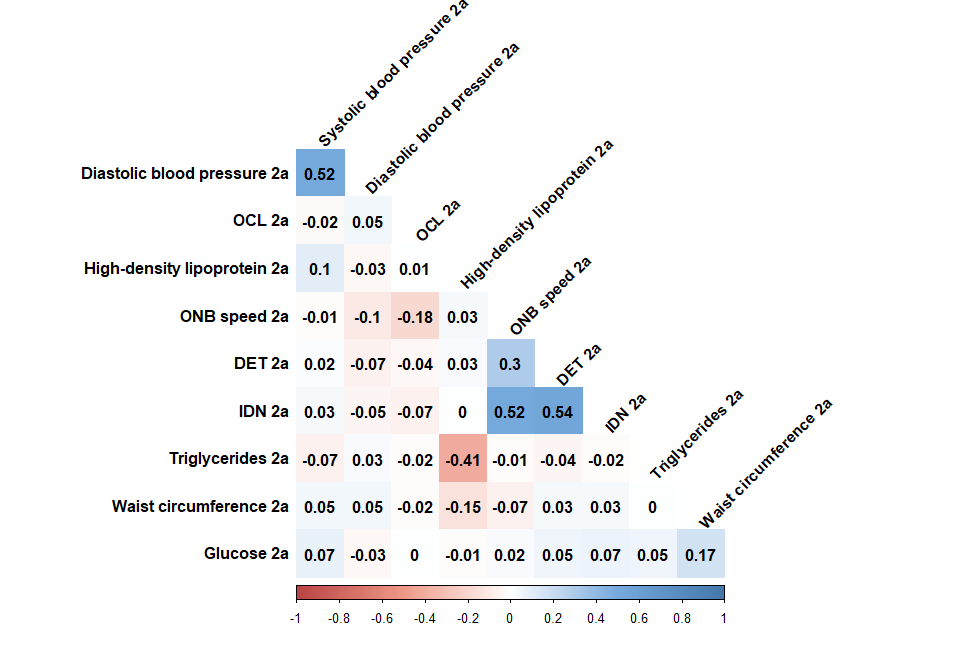

Supplement: Supplementary file 1 — Supplementary material, approximately 661 KB. [file 42414_2024_352_MOESM1_ESM.docx]
